# Complementary information concerning the suspected interindividual transmission of GW1516, a substance prohibited in sport, through intimate contact: a case report

**DOI:** 10.1007/s11419-024-00689-x

**Published:** 2024-05-05

**Authors:** J. Breuer, A. M. Garzinsky, A. Thomas, E. Nieschlag, S. Kliesch, M. Fedoruk, H. Geyer, M. Thevis

**Affiliations:** 1 Institute of Biochemistry, Center for Preventive Doping Research, German Sport University Cologne, Am Sportpark Müngersdorf 6, 50933 Cologne, Germany; 2 University Hospital Muenster (UKM), Muenster, Germany; 3 Science and Research, USADA, Colorado Springs, USA; 4 European Monitoring Center for Emerging Doping Agents (EuMoCEDA), Cologne, Bonn Germany

**Keywords:** Seminal fluid, Exposome, Sports, Doping, GW1516

## Abstract

**Purpose:**

Inadvertent and/or unknowing exposure to drugs and drug residues has been frequently debated in situations of so-called adverse analytical finding (AAF) in the context of sports drug testing programs. Transfer of drug residues via unprotected intercourse is a conceivable scenario but scientific data and authentic case reports are scarce. Herein, investigations into two AAFs with the peroxisome proliferator-activated receptor delta (PPARδ) agonist GW1516 are reported and discussed.

**Methods:**

To probe for a contamination scenario involving sexual intercourse, two assays were used to determine semenogelin in human urine, with one employing an immunochromatographic lateral flow approach and another based on liquid chromatography–tandem mass spectrometry. Further, drug-residue testing using patients’ ejaculate was conducted by utilizing liquid chromatography in conjunction with a triple quadrupole mass spectrometer, followed by re-analysis of suspect samples (i.e., samples indicating the presence of relevant compounds) using high resolution/high mass accuracy mass spectrometry.

**Results:**

In one case, but not the other, the possibility of intimate contact as the source of the AAF was confirmed after a thorough investigation of potential contamination scenarios. Subsequent research revealed analytical evidence for the presence of seminal fluid in one of the female athlete’s doping control urine samples, and the analysis of clinical ejaculate specimens provided first data on an authentic concentration level of GW1516 and its metabolites in human seminal fluid.

**Conclusions:**

The combined facts substantiate the possibility of an AAF caused by unprotected sexual intercourse and the plausibility of the case-related arguments.

## Introduction

A central topic of anti-doping research has been the growing necessity of distinguishing scenarios of intentional doping from situations commonly referred to as contamination or unknowing exposure. A major challenge in that context is the complex environmental influence, which can affect an athlete’s career considerably more serious than the general population due to their participation in sports drug testing programs. Prohibited substances can be transferred in trace amounts to athletes, which can then lead to an adverse analytical finding (AAF) [1]. The detection of minute concentrations of doping agents has been of utmost importance to doping controls, and the evolution of analytical techniques has offered increasing sensitivities that allow for the required analytical retrospectivity in sports drug testing. However, distinguishing between intentional drug use (i.e., doping) committed some time ago and trace contamination has become more and more difficult. One frequently discussed scenario of drug exposure in the context of AAFs is the transmission of substances through intimate contact such as sexual intercourse, which was accepted as early as 2004 as a possible contamination scenario [2].

Non-threshold doping substances (prohibited in- and out-of-competition) classified in the World Anti-Doping Agency (WADA) Prohibited List without a so-called minimum reporting limit (MRL) are of particular concern as their detection and confirmation results in an AAF regardless of their concentration [3].

One example is GW1516 (also known as GW501516 or Cardarine), a peroxisome proliferator-activated receptor delta (PPARδ) agonist [4, 5]. The PPARδ in skeletal muscle cells has distinct roles in the regulation of lipid, carbohydrate, and energy homeostasis. Studies have shown that the AMPK-PPARδ pathway can be influenced by orally administered drugs such as GW1516 to improve exercise adaptation or even increase athletic endurance performance without training [6]. GW1516 was discussed as an emerging doping agent in 2008 and first explicitly mentioned in the WADA Prohibited List in 2009 as a hormone and metabolic modulator [7]. Clinical trials with GW1516 were discontinued when animal studies demonstrated carcinogenic effects [8].

However, even though GW1516 is not commercially available as an approved drug, 139 AAFs involving GW1516 have been reported to WADA since 2009. The number of cases varies widely, from a minimum of 1 (2012) to a maximum of 31 (2016) cases per year. Overall, GW1516 AAFs account for 1–12% of cases in substance class S4 [9].

There are several reports describing the possibility of doping control samples containing prohibited substances because of intimate contact [10–13], but little (if any) information has been presented on analytical options in support of follow-up investigations and result management. In the herein presented two cases, the combined information from routine doping controls, follow-up analyses, and new data on drug levels in seminal fluid provided a dataset plausibly and convincingly supporting the claimed scenario of contamination.

## Case reports

A urine sample from a female athlete that returned low but detectable amounts of the metabolites of GW1516 (GW1516 sulfoxide estimated at 4 pg/mL and GW1516 sulfone estimated at 5 pg/mL) and LGD-4033 metabolite was reported as an AAF. The doping control sample was taken out-of-competition, and the athlete claimed to have had unprotected sexual intercourse on the day of sample collection. Based on a variety of factors, the athlete did not face any period of ineligibility. A urine sample from a second female athlete that returned low but detectable amounts of the metabolites of letrozole (bis(4-cyanophenyl)methanol) and GW1516 sulfoxide estimated at 14 pg/mL and GW1516 sulfone estimated at 5 pg/mL. The doping control sample was taken out-of-competition, and the athlete claimed to have had unprotected sexual intercourse on the day of sample collection. Investigations revealed that her partner had taken oral solutions of letrozole and GW1516 on a daily basis for a period of 2 to 3 weeks. Based on credible testimony, the athlete did not face any period of ineligibility.

## Material and methods

### Chemicals and reagents

Acetonitrile (ACN), methanol and formic acid were obtained from VWR chemicals (Langenfeld, Germany). Ammonium acetate and acetic acid were obtained from Merck (Darmstadt, Germany). Ultrapure water was received from a Barnstead GenPure xCAD Plus from Thermo Scientific (Bremen, Germany). The Rapid Stain Identification (RSID™)-semen field test was obtained from Independent Forensics (Lombard, IL). The seminal fluid samples were collected as part of routine andrological examinations at Muenster University Hospital (Germany) and consent was obtained for further research purposes.

### Probing for the presence of semenogelin by lateral flow immunochromatographic analysis

To test for the presence of semenogelin, a lateral flow immunochromatographic analysis was performed as an initial-testing procedure as described by Breuer et al., 2021 [13]. In brief, 20 µL of urine was diluted with 80 µL RSID™ Universal Buffer and transferred on the lateral flow immune strip. Positive and negative controls were prepared and analyzed as recommended. Ten minutes after application, the result (presence or absence of semen) was photographically documented.

### Probing for the presence of semenogelin I by liquid chromatography–tandem mass spectrometry (LC–MS/MS)

The LC–MS/MS analysis was performed as described by Breuer et al., 2021 [13]. Briefly, after preparation of the urine samples by solid-phase extraction, tryptic digestion was performed. The analysis for the presence of three specific peptides of semenogelin I was conducted using a Vanquish™ UHPLC system coupled to a Thermo Scientific Orbitrap Exploris™ 480 mass spectrometer (Thermo Scientific, Bremen, Germany). Characteristics of peptides of semenogelin and the internal standard (ISTD) used for the LC–MS/MS assay are listed in Table 1.

**Table 1:** Characteristics of peptides of semenogelin I and the internal standard (hemoglobin) used in the LC–MS/MS assay [13]

Analyte	Peptide	Amino-acid sequence	Sum formula	Precursor ion (*m/z*)	Qualifier ion (*m/z*)	Qualifier ion (*m/z*)	NCE (%)
Semenogelin I (human)	T21	GTQNPSQDQGNSPSGK	C_62_H_10_N_22_O_28_	801.36	1201.54	388.22	25
	T46	QITIPSQEQEHSQK	C_69_H_113_N_21_O_26_	826.92	1197.55	214.16	30
	T57	EQDLLSHEQK	C_51_H_83_N_15_O_20_	613.8	628.31	741.39	25
Hemoglobin (bovine)	T6	FFESFGDLSTA1DAVMNNPK	C_93_H_136_N_22_O_31_S	1045.48	1147.55	1432.7	30

### Analysis of GW1516 in seminal fluid: sample preparation and extraction procedure

To 100 µL of seminal fluid, 5 μL of an ISTD working solution was added and mixed with 100 µL acetic acid (1%). To precipitate proteins, 800 µL of ice-cold ACN was added, and the sample was vortexed for 20 s and cooled for 20 min at 4°C. Following centrifugation (15 min, 14,000 × *g*), the pellet was discarded and the supernatant was evaporated to dryness in a vacuum centrifuge. The dried sample was reconstituted in 100 μL of H_2_O/ACN (80/20, *v/v*).

### Analysis of GW1516 in seminal fluid: LC–MS/MS instrumentation and analytical conditions: initial-testing procedure

LC–MS/MS analysis of seminal fluid was conducted using an Aquity I-Class ultra-performance liquid chromatograph (UPLC) coupled to a Xevo Triple Quadrupole-XS mass spectrometer (TQ-MS), both from Waters (Eschborn, Germany). Seminal fluid samples prepared for analysis were injected onto a Poroshell C-8 analytical column (50 × 3.0 mm, 2.7 μm particle size; Agilent, Waldbronn, Germany) employing the eluents A (0.1% formic acid in water) and B (0.1% formic acid in ACN) at a flow rate of 0.4 mL/min. The method started at 5% B, increasing to 15% B from 1 to 2 min, followed by an increase to 55% B from 2 to 11 min. B was then increased to 100% from 11 to 12 min, followed by equilibration at 1% B from 12 to 15 min. Before the next injection, the needle was washed for 1 min while B was increased from 1% to 5%. The compounds were introduced into the mass spectrometer by electrospray ionization and were detected by time-based multiple-reaction monitoring experiments. The data were processed using TargetLynx™ (Waters, Eschborn, Germany) after analysis.

### Analysis of GW1516 in seminal fluid: Liquid chromatography–high resolution mass spectrometry (LC–HRMS) instrumentation and analytical conditions: confirmation procedure

A LC–HRMS method was developed for the analysis of GW1516 and its metabolites. The LC–HRMS system used was a Vanquish HPLC system coupled to a Thermo Scientific Orbitrap Exploris™ 480, both from Thermo Scientific (Bremen, Germany). For chromatography, a Poroshell 120 EC-C18 analytical column (50 × 3.0 mm, 2.7 μm particle size; Agilent, Waldbronn, Germany) was used, connected to an EC 4/3 Nucleoshell RP 18 Plus guard column (4 × 3 mm, 5 μm particle size) from Macherey–Nagel (Düren, Germany). The gradient with a run time of 15 min and a flow rate of 0.4 mL/min was performed using water containing 0.1% acetic acid as solvent A and ACN containing 0.1% acetic acid as solvent B. The method was started with 0% B, increasing to 90% B in 11 min. Subsequently, 100% B was held for 1 min followed by re-equilibration at 0% B for 3 min. The injection volume was 5 µL. Measurements were conducted in positive ionization mode with an ionization voltage of 3 kV and a transfer tube temperature of 320 °C. A resolution of 60,000 full width at half maximum (FWHM) was selected for the full-scan and 45,000 FWHM for HRMS/MS experiments, and the full-scan was performed in a range of *m/z* 100–800. The normalized collision energy (NCE) and precursor ions are listed in Table 2. For product ion scan experiments, the isolation window of the quadrupole was set to 1 *m/z*. Nitrogen was generated by the CMC nitrogen generator (Eschborn, Germany) and used as collision gas. The Thermo Scientific Orbitrap Exploris™ 480 was calibrated regularly with the manufacturer’s calibration solution.

**Table 2:** Characteristics of GW1516, GW1516-Sulfoxide, GW1516-Sulfone and the internal standard used in the LC–HRMS-confirmation assay

Analyte	Sum formula	Precursor ion (*m/z*)	Product ion (*m/z*)	Product ion (*m/z*)	NCE (%)	RT (min)
GW1516	C_21_H_19_O_3_NF_3_S_2_	454.0765	257.0477	188.0525	50	10.23
GW1516-Sulfoxide	C_21_H_19_O_4_NF_3_S_2_	470.0709	257.0477	188.0525	50	8.01
GW1516-Sulfone	C_21_H_19_O_5_NF_3_S_2_	486.0658	257.0477	188.0525	50	8.70
Stanozolol-*d*_3_ (ISTD)	C_21_H_30_N_2_OD_3_	332.2775	81.0447		65	6.87

*RT* retention time

## Results

**Fig. 1:**
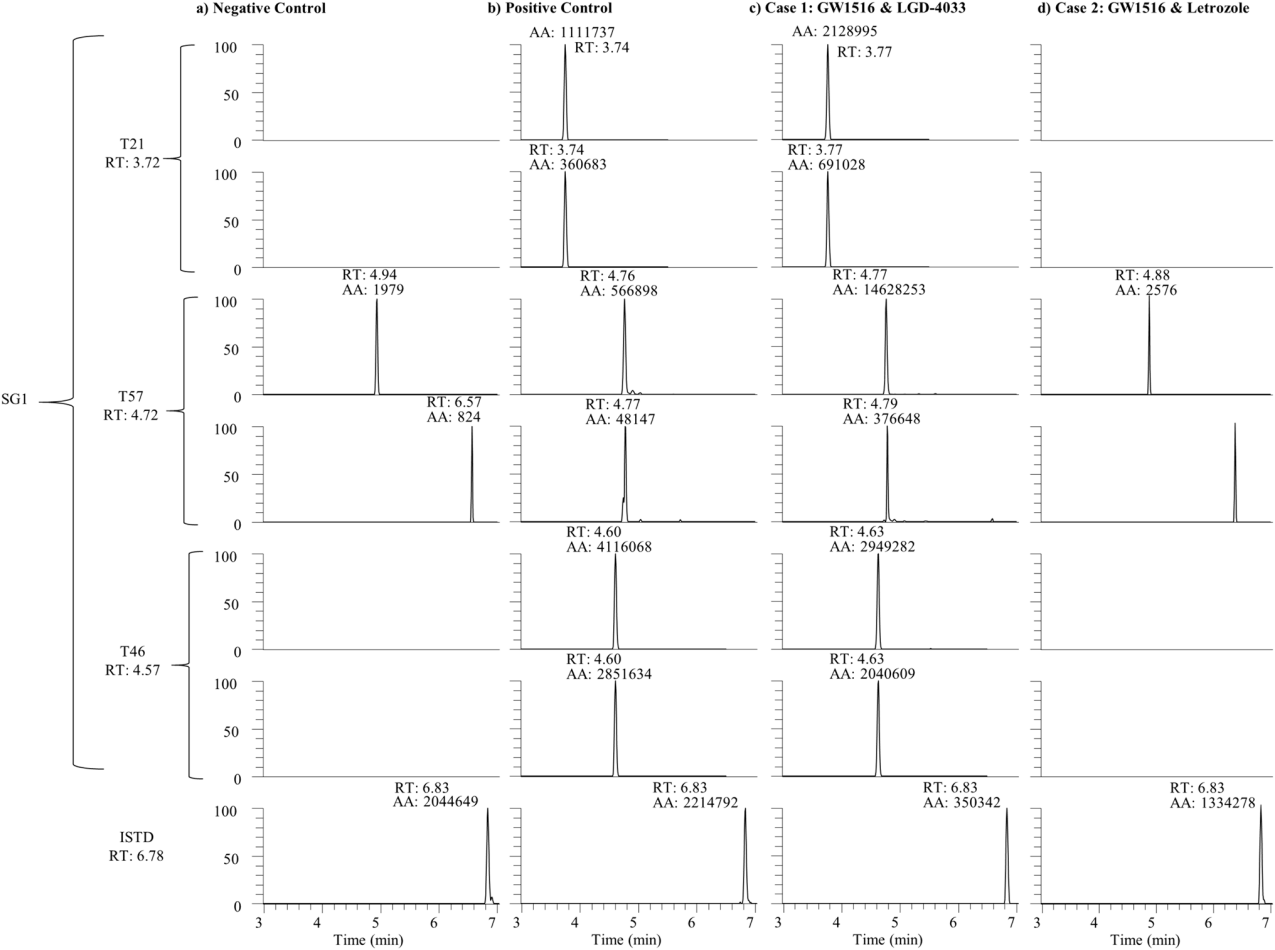
Extracted-ion chromatograms of a blank urine (**a**), a urine specimen containing 100 nL of semen (**b**), the authentic doping control urine sample containing GW1516 and LGD-4033 metabolites (**c**) and the authentic doping control urine sample containing GW1516 and letrozole metabolites (**d**)

After the publication of a method for the detection of semenogelin [13], a protein specific for seminal fluid and thus a marker for unprotected sexual intercourse, an aliquot of the urine sample from the cases described above was requested for retrospective analysis. The initial-testing procedure by a laminar flow immunologic test strip method yielded a positive result of semenogelin for one sample (GW1516 metabolites/LGD-4033 metabolite), but not the other sample (GW1516 metabolites/letrozole metabolite) (data not shown). The presence of semenogelin in the urine sample was confirmed by the detection of three specific peptides in MS/MS experiments (see Table 1 and Fig. 1). Both analytical results thus showed that seminal fluid was present in the urine sample and, therefore, confirmed the possibility that GW1516-containing seminal fluid was a potential contaminant of the athlete’s doping control urine sample. In addition, the results confirm the athlete’s statement that she had intimate contact within hours prior to the sample collection. However, the absence of semenogelin in the sample of case 2, despite the clear evidence of intimate contact on the day of sample collection suggest an important limitation of relying solely on a semen-exposure marker as definitive evidence of intimate contact, as due to female anatomy, it is reasonable to not always expect semen-contamination of a female urine doping control sample post-coitus.

To probe for the potential and extent of a transfer of drugs into seminal fluid, a total of 361 seminal fluid samples was obtained from the University Hospital in Münster (Germany). The specimens were analyzed to determine the concentration of therapeutics and illicit substances classified as doping agents in the general population (publication pending). Therefore, a screening method was developed using a TQ-MS to test the samples for 56 different target analytes, and samples suspected to contain one or more doping agents were further subjected to an LC–HRMS/MS confirmation procedure to probe for the presence and quantity of the target analyte and potential (example for GW1516 shown in Table 2).

**Fig. 2:**
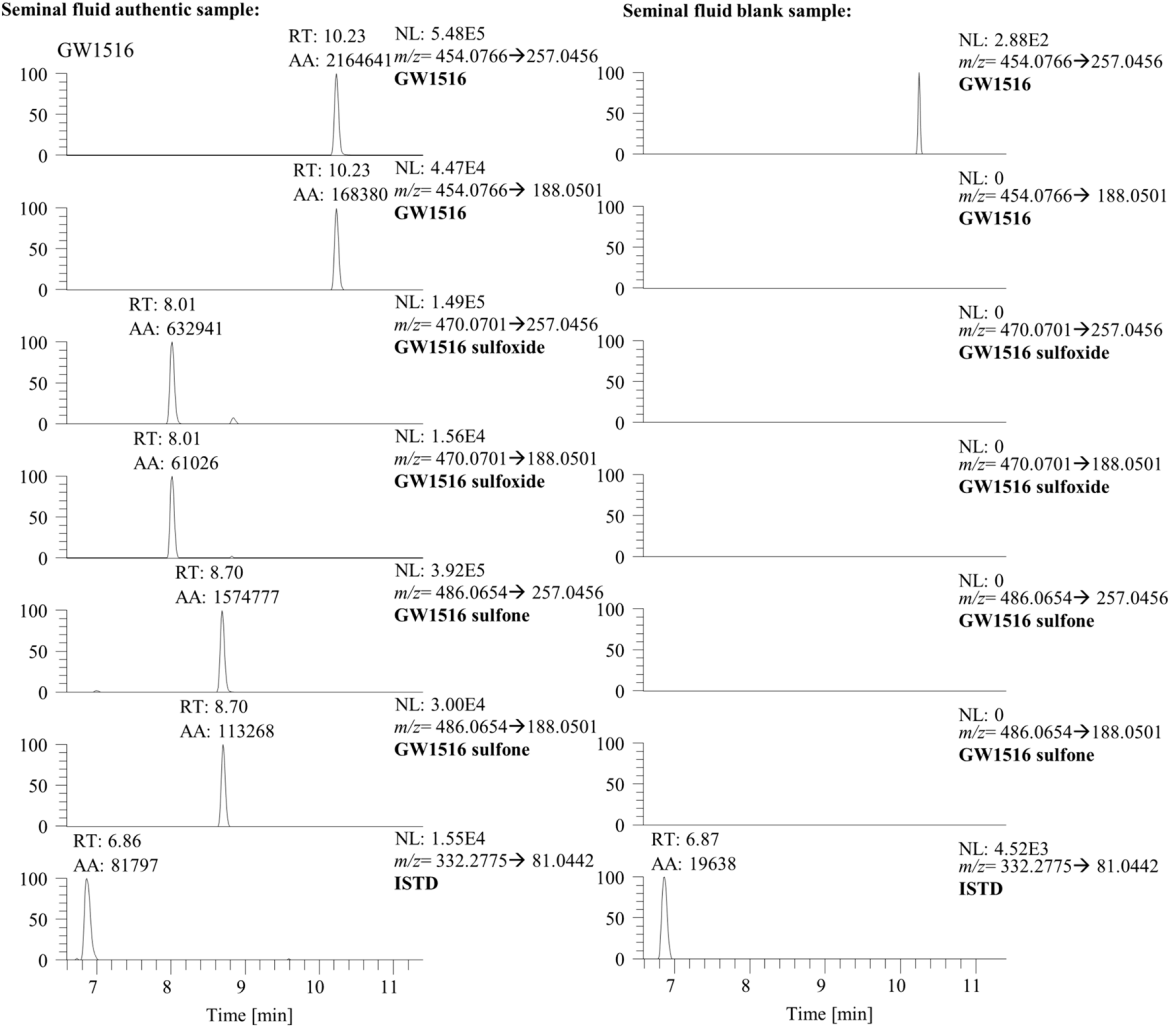
Extracted-ion chromatograms of the authentic seminal fluid sample containing GW1516 and GW1516 metabolites (left) and a blank seminal fluid sample (right)

One of these investigated seminal fluid samples was found to contain GW1516. Using the confirmatory method, the concentration of GW1516 was determined to be approximately 48 ng/mL. In addition, the metabolites GW1516 sulfoxide and GW1516 sulfone (Fig. 2) were detected. Although the administered amount of GW1516 is unknown, these results show that high concentrations of GW1516 can occur in seminal fluid and could lead to an AAF, even when considering the dilution factor in urine.

## Discussion

These case reports provide a dataset that plausibly supports the scenario of seminal fluid as a possible source of contamination leading to an AAF. The estimated concentration of GW1516 sulfoxide and sulfone were 4 pg/mL and 5 pg/mL, respectively; the athlete’s doping control urine sample was proven to contain semenogelin I (a seminal fluid-specific protein), and GW1516 (and its metabolites) was shown to transfer into seminal fluid as demonstrated by means of a clinical sample.

Yet, several aspects of this case remain unknown (e.g., the presumed concentration of GW1516 in the athlete’s partner’s ejaculate, the volume of ejaculate potentially contained in the doping control urine sample) and, hence, the AAF cannot exclusively be attributed to a contamination scenario [13]. Hence, additional aspects assisting in assessing the likelihood of the scenario should be factored-in, such as the average volume of 3.7 mL of seminal fluid [14] transferred during sexual intercourse and the minimum volume of 90 mL of urine collected in a routine doping control setting according to WADA regulations.

When applying the GW1516 concentration observed in the clinical sample to the athlete’s doping control scenario, a 1/100 fraction (i.e., 37 µL) of seminal fluid transmitted during sexual intercourse and introduced into the doping control urine sample would theoretically suffice to produce the reported AAF.

The interindividual transfer of drugs has also been described in other situations. For instance, in a recent case report, the absorption of drugs into the cervical mucosa via seminal fluid was described [15]. There, severe symptoms corresponding to acute opioid withdrawal occurred after sexual intercourse. This adds another layer of complexity, namely that not only are substances transmitted during sexual intercourse and can lead to possible contamination by introduction of (drug-containing) seminal fluid into the doping control urine specimen, but they can also be absorbed, metabolized, and eliminated.

## Conclusions

The herein presented data support the assumption that doping control urine samples can be contaminated with doping agents caused by sexual intercourse. The detection of semenogelin in a doping control sample that returned an AAF for GW1516 confirmed the intimate contact claimed by the athlete. Semenogelin presence can be used as strong supporting evidence for prohibited-substance exposure via the introduction of male ejaculate contaminating a female athlete’s doping control urine sample, however there are some important limitations, therefore the presence (or absence) cannot be used as unequivocal evidence of prohibited-substance exposure via sexual transmission. It has also been shown that doping agents such as GW1516 can be present in seminal fluid in concentrations sufficient to cause an AAF when introduced into a female athlete’s urine at volumes of 37 µL, and that commonly observed GW1516 metabolites are also present in seminal fluid when GW1516 is used. Further studies on the concentrations and metabolite profiles of prohibited substances, which also have a relevance for therapeutic use in the general population, are important to be able to provide the data required for assessing probabilities of debated scenarios.

Complementary matrices have also been suggested to contribute information for case management, e.g., hair testing of the athlete and/or the partner (allegedly) using the prohibited substance [11]. However, also here analytical as well as legal issues might apply, as the person who is not in the doping control system and asked to provide the hair sample might refuse to do so or no hair segments covering the relevant time period are available.

## Data Availability

The datasets generated and analyzed as part of this study are available upon request from the corresponding author.
